# DR5 Up-Regulation Induced by Dichloroacetate Sensitizes Tumor Cells to Lipid Nanoparticles Decorated with TRAIL

**DOI:** 10.3390/jcm12020608

**Published:** 2023-01-12

**Authors:** Joaquín Marco-Brualla, Diego de Miguel, Luis Martínez-Lostao, Alberto Anel

**Affiliations:** 1 Apoptosis, Immunity and Cancer Group, Department of Biochemistry and Molecular and Cell Biology, Aragon Health Research Institute (IIS-Aragón) & University of Zaragoza, 50009 Zargoza, Spain; 2 Immunology Department, Lozano Blesa Clinical Hospital, 50009 Zaragoza, Spain

**Keywords:** TRAIL, LUV-TRAIL, dichloroacetate, DR5

## Abstract

Cancer resistance to treatments is a challenge that researchers constantly seek to overcome. For instance, TNF-related apoptosis-inducing ligand (TRAIL) is a potential good prospect as an anti-cancer therapy, as it attacks tumor cells but not normal cells. However, treatments based in soluble TRAIL provided incomplete clinical results and diverse formulations have been developed to improve its bioactivity. In previous works, we generated a new TRAIL formulation based in its attachment to the surface of unilamellar nanoliposomes (LUV-TRAIL). This formulation greatly increased apoptosis in a wide selection of tumor cell types, albeit a few of them remained resistant. On the other hand, it has been described that a metabolic shift in cancer cells can also alter its sensitivity to other treatments. In this work, we sought to increase the sensitivity of several tumor cell types resistant to LUV-TRAIL by previous exposure to the metabolic drug dichloroacetate (DCA), which forces oxidative phosphorylation. Results showed that DCA + LUV-TRAIL had a synergistic effect on both lung adenocarcinoma A549, colorectal HT29, and breast cancer MCF7 cells. Despite DCA inducing intracellular changes in a cell-type specific way, the increase in cell death by apoptosis was clearly correlated with an increase in death receptor 5 (DR5) surface expression in all cell lines. Therefore, DCA-induced metabolic shift emerges as a suitable option to overcome TRAIL resistance in cancer cells.

## 1. Introduction

TNF-related apoptosis-inducing ligand (TRAIL) belongs to the TNF superfamily. Its potential to specifically trigger tumor cell death has been widely demonstrated, inducing apoptosis in a plethora of tumor types [1,2,3] or necroptosis if there is caspase inhibition [4,5] since its discovery. TRAIL can recognize several membrane-bound receptors, which are: TRAIL-R1 (DR4), TRAIL-R2 (DR5), TRAIL-R3 (DcR1), and TRAIL-R4 (DcR2), as well as soluble circulating osteoprotegerin (OPG). Among these proteins, only DR4 and DR5 have proven to transduce TRAIL-induced death signaling, whereas the other membrane receptors act as decoys, as they do not possess intracellular death domains [6].

TRAIL binding to their receptors is followed by its homotrimerization [7,8]. This event triggers oligomerization of the intracellular death domains and the subsequent recruitment of Fas-associated death domain (FADD), an adapter protein. FADD recruitment also induces the arrival and binding of procaspase-8 and other proteins, such as the cellular inhibitor of apoptosis proteins (cIAPs) and the receptor-interacting serine/threonine-protein kinase 1 (RIPK1), merging into the death-inducing signaling complex (DISC). This complex can promote the cleavage of caspase-8, which is thus activated and free in the cytosol to cleave effector caspases-3 and -7, finally triggering apoptosis in the cell [9]. From the two death-inducing TRAIL receptors, it has been described at least in several epithelial tumor types and in leukemic cells that DR5 possesses superior death-inducing potency [10,11]. On the other hand, the protein c-FLIP can prevent the extrinsic pathway of apoptosis by inhibiting DISC formation [12,13]. TRAIL can also activate the intrinsic mitochondrial apoptotic pathway through caspase-8-mediated cleavage of Bid, a BH3-only pro-apoptotic member of the Bcl-2 family [14]. The balance between the expression of pro-apoptotic or anti-apoptotic members of the BCL2 family can also influence the final outcome of death receptor ligation in tumor cells. Bim is one of the most important pro-apoptotic members of this family, while Bcl-x_L_, Mcl-1, or Bcl-2 are prominent anti-apoptotic members of the family [15].

Since its identification, TRAIL has been established as an interesting choice for cancer treatment, as it targets tumor cells while not damaging normal cells [16]. Nevertheless, clinical trials using soluble TRAIL or agonists of TRAIL receptors have been rather disappointing, showing poor tumor responsiveness overall, despite promising preclinical results [17]. This lack of clinical efficacy can be due not only to tumor intrinsic or acquired resistance to TRAIL, but also because these TRAIL agonists were just not potent or stable enough.

In order to improve TRAIL effectiveness, research has been focused on TRAIL formulations with higher bioactivity or half-life in patients [17]. Physiologically, TRAIL is found in the lipid bilayer as a transmembrane protein [8,18]. In addition, it was also demonstrated that TRAIL can be secreted from activated T lymphocytes in association with exosomes once they are activated. Importantly, this exosome-bound TRAIL not only conserved its pro-apoptotic potential, but it presented increased bioactivity over soluble recombinant TRAIL [19,20]. In connection with this, the anchorage on a membrane improves TRAIL stability and cross-linking, which can be exploited for cancer therapy [1]. Following these observations, and aiming to imitate the exosome-bound TRAIL, we successfully created, in previous works, artificial lipid nanoparticles containing TRAIL on their surface (LUV-TRAIL) with a higher in vitro cytotoxicity against several types of cancer cell lines, in comparison with soluble recombinant TRAIL, both in vitro and in vivo [10,21,22,23,24,25,26].

Apart from this, combination therapies enhancing TRAIL anti-tumor activity have also been developed, with positive pre-clinical results. For example, TRAIL can synergize with proteasome inhibitors [27,28], radio- or chemotherapy [29], SMAC mimetics [30], or metabolic drugs [31,32]. Among them, the latter is one of the best options to put into consideration. Remodeling of tumor energetic metabolism is a common characteristic of cancer cells [33] as they usually change their preference of obtaining energy from oxidative phosphorylation (OXPHOS) to glycolysis and lactic fermentation, even in the presence of oxygen; a phenomenon known as the Warburg effect [34]. Despite being a less efficient process, this pathway allows tumor cells to obtain energy faster than through OXPHOS [35]. Therefore, this particular feature makes energy metabolism an exploitable target for cancer therapy.

Dichloroacetate (DCA) is a metabolic drug that inhibits pyruvate dehydrogenase kinase (PDK), forcing the pyruvate dehydrogenase complex to remain active, and thus promoting the conversion in the mitochondria of pyruvate into acetyl-CoA and the use of OXPHOS. Its use against cancer began to raise interest in the last decade [36] and several phase-1 clinical trials have been made since then [37,38,39]. In summary, it seems that DCA is able to directly kill tumor cells with defects in their respiratory chain [40,41]; however, if that is not the case, it mainly works as a cell growth inhibitor or as a sensitizing agent. In fact, this compound has proven to synergize with multiple anti-cancer agents, such as cisplatin [42], doxorubicin [43], or metformin [44]. From an immunological standpoint, DCA is able to increase the expression of the MHC-I, sensitizing tumor cells to the action of cytotoxic T lymphocytes [45], and also to increase the expression of the NK cell activating receptors MICA/B and ULBP1, as well as ICAM-1 [46]. In fact, it has been demonstrated at least in one study that the combination of DCA and metformin are able to sensitize the mammary adenocarcinoma MCF7 to cell death induced by soluble TRAIL through upregulation of DR5 expression [47]. However, in that study it was not determined if this effect was due to DCA or to metformin.

In this work, we explored the sensitizing ability of DCA to the cytotoxic action of the LUV-TRAIL formulation and characterized the possible mechanisms behind this combinatory effect.

## 2. Materials and Methods

### 2.1. Cells and Cell Culture

The non-small cell lung cancer (NSCLC) A549 cell line as well as the colorectal cancer HT29 cell line were purchased in ATCC and the breast cancer MCF7 cell line was kindly provided by Dr. Abelardo López Rivas.

All cell lines were cultured in high glucose DMEM (Gibco, Waltham, MA, USA) medium supplemented with 10% heat-inactivated FBS (Sigma, St. Louis, MO, USA), penicillin/streptomycin (Pan Biotech, Aidenbach, Germany) and GlutaMAX (Gibco, Waltham, MA, USA).

### 2.2. Formation of Lipid Nanoparticles Anchored with Soluble Recombinant TRAIL

Generation of large unilamellar vesicles (LUV) with coated human soluble recombinant TRAIL (sTRAIL) was carried out as explained in previous works [21,48]. Briefly, a mix of several lipids were prepared in a weight ratio of 50:30:10:5:5: phosphatidylcholine, sphingomyelin, cholesterol, 1,2-distearoyl-sn-glycero-3-phosphoethanolamine)-N-(methoxy(polyethylene glycol)-2000) (ammonium salt), and 1,2-dioleoyl-sn-glycero-3-[N-(5-amino-1-carboxypentyl)-iminodiacetic acid] succinyl (nickel salt). All of them were purchased from Avanti Polar Lipids, Alabaster, AL, USA. Next, chloroform was removed by drying the mixture: firstly, by exposure of nitrogen gas; and secondly, under a vacuum at 37 °C. After this, lipids were hydrated in phosphate buffer saline (PBS). Liposomes of one layer were finally obtained by two steps: freeze thawing (at least 2 times) and extruding (at least 10 times) through two 200 nm pore-sized polycarbonate membranes (Whatman, Maidstone, UK) using an extruder (Northern Lipids, Burnaby, Canada). Z-average was of 155 nm with a width of 61.2 nm for LUVs alone, while in the case of LUVs coated with TRAIL it was of 178 nm with a width of 85.3 nm. This was also confirmed by scanning electron microscopy, in which vesicles with a diameter from 150 to 200 nm were observed, with a high degree of homogeneity [21].

TRAIL was bound to LUVs by incubation of liposomes for 30 min at 37 °C with sTRAIL-His6 (amino acids 95-281, kindly provided by Dr. M. MacFarlane) [49]. The efficient coating of the particle by TRAIL was characterized by biophysical determinations in the first studies in which these nanoparticles were described, with a 95% recovery of the protein bound to the liposomes [21,48].

### 2.3. Cell Incubation with Drugs

After counting and culturing tumor cells in the appropriate wells, they were incubated with the drugs indicated. Cells were incubated with DCA (Sigma, St. Louis, MO, USA) at different concentrations, from 5–25 mM, for 24–72 h. Every 48 h, DCA was renewed. For the drug combination experiments, after 48 h or 72 h incubation with DCA, LUV-TRAIL (1000 ng/mL) was added for another 24 h before analysis of results. In some experiments, the general caspase inhibitor carbobenzoxy-valyl-alanyl-aspartyl-[O-methyl]- fluoromethylketone (abbreviated as z-VAD) (MedChemExpress, Monmouth Junction, NJ, USA) or the specific TRAIL blocking antibody RIK2 (BD Biosciences, Franklin Lakes, NJ, USA) were added to tumor cells.

### 2.4. Flow Cytometry Assays

For both assays, a FACSCalibur flow cytometer was used to detect the cells and a computer with the CellQuestPro software (BD Biosciences, Franklin Lakes, NJ, USA) was used to collect the data. Afterwards, Flow Jo software (Tree Star Inc., San Francisco, CA, USA) was used to analyze them.

DR4 and DR5 surface expression was determined as follows: 1 × 10^5^ tumor cells were incubated with specific antibodies for DR4, DR5, DcR1, or DcR2. An additional isotype control antibody was also used (all of them conjugated with PE and purchased from eBioscience, San Diego, CA, USA). Staining conditions were: tumor resuspension in PBS containing 5% FBS with the corresponding antibody for 30 min at 4 °C. Then, cells were washed once, resuspended in 300 µL with PBS and analyzed in the FACSCalibur.

For the measurement of cell death induction, 1 × 10^5^ tumor cells were incubated for 15 min with both Annexin-V-FITC (Immunostep, Salamanca, Spain), which recognizes exposed phosphatidylserine, and 7-aminoactinomycin D (7-AAD, Biolegend, San Diego, CA, USA), which binds DNA. The mixture was resuspended in annexin-binding buffer (140 mM NaCl, 2.5 mM CaCl_2_, 10 mM HEPES/NaOH, pH 7.4). After staining, cells were washed once with PBS and their fluorescence was measured by flow cytometry.

### 2.5. Cell Growth Assay

Relative cell proliferation was assessed by the Mosmann modified method for microplates [50]. In brief, 10^4^ cells/mL were cultured in 96-well flat-bottomed plates. Following cell attachment to the wells, tumor cells were incubated with DCA and/or LUV-TRAIL at the indicated concentrations and times. When drug exposition was finished, MTT dye solution (10 μL of 5 mg/mL of 3-(4,5-dimethylthiazol-2-yl)-2,5 diphenyltetrazolium bromide, in PBS) was added to each well. After 2–3 h incubation, cells that remained alive during drug treatment processed the MTT and formed purple formazan crystals. These insoluble precipitates were resuspended in isopropanol. Color from each experimental point was measured by absorbance at 550 nm in a microplate reader (Dynatech, Pina de Ebro, Spain). Results were normalized to untreated cells and expressed as a percentage of growth.

### 2.6. Western-Blot Experiments

The expression of several proteins from cell extracts was determined by Western blot. For each experiment condition, at least 3 × 10^6^ cells were centrifuged and lysed in 30 μL per million in the following solution: buffer containing 10 mg/mL leupeptin, 1 mM PMSF, 10% glycerol, 0.15 M NaCl, 1 mM EDTA, and 1% Triton X-100 in 50 mM Tris/HCl (pH 7.4) distilled water. After 30 min of lysis at 4 °C, cell membranes were discarded by centrifugation and protein suspensions from cells were loaded in a SDS-12% polyacrylamide gel. Proteins were separated by gel electrophoresis and then transferred to PVDF membranes. These membranes were blocked with PBS-T buffer (10 mM Tris/HCl pH 8.0; 0.12 M NaCl; 0.1% Tween-20) containing 5% skimmed milk. PVDF membranes were incubated with one of the following antibodies: anti-c-FLIP (Enzo Life Sciences, Farmingdale, NY, USA), anti-Bim (Calbiochem, San Diego, CA, USA), anti-Bcl-xL (Cell Signaling, Danvers, MA, USA), anti-Bcl-2 (Abcam, Cambridge, UK), anti-Bid (R&D Systems, Minneapolis, MN, USA), anti-caspase 3 (R&D Systems, Minneapolis, MN, USA), anti-Mcl-1 (Santa Cruz Biotech, Dallas, TX, USA), or anti-XIAP (BD Transduction, Franklin Lakes, NJ, USA) in an antibody solution (PBS-T + 5% *w*/*v* bovine serum albumin and 0.05% *w*/*v* NaN_3_). Membranes were washed several times with PBS-T and then exposed to 0.2 μg/mL of the corresponding peroxidase-labeled antibody (Sigma, St. Louis, MO, USA). Protein expression was revealed by using the Pierce ECL Western Blotting Substrate (Thermo Scientific, Waltham, MA, USA)

### 2.7. Statistical Analysis

Statistical tests were analyzed using the GraphPad Prism 8 program (GraphPad Software Inc., San Diego, CA, USA). Error bars in the graphics reflect the +/− SD of the mean value of the experiments. Student’s *t* test for paired variants was performed to assess differences between data. A value of *p* < 0.05 was accepted as significant in all cases.

## 3. Results

### 3.1. DCA Sensitizes Tumor Cells to LUV-TRAIL Treatment

Lung adenocarcinoma A549 cells, colorectal cancer HT 29 cells, and breast cancer MCF7 cells were chosen for this experiment, as they had previously proven not to be highly sensitive to LUV-TRAIL treatment alone. As a first approach to check DCA ability to synergize with LUV-TRAIL, tumor cells were incubated for either 48 h or 72 h with 25 mM DCA. Afterwards, LUV-TRAIL was added for another 24 h. Finally, cell growth and apoptosis induction were measured. Additionally, basal cell death of cancer cells alone, either with DCA (24–96 h) or with LUV-TRAIL (24 h), was also assessed.

**Figure 1:**
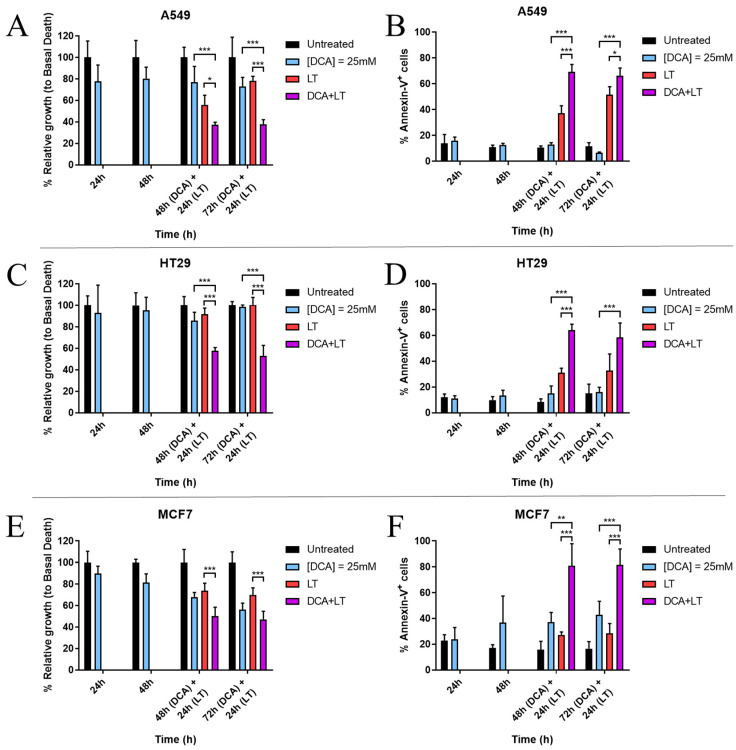
Effect of DCA + LUV-TRAIL (LT) combination in cell growth (MTT assay) and cell death (annexin-V staining and detection by flow cytometry) in (**A**,**B**) A549, (**C**,**D**) HT29, and (**E**,**F**) MCF7 cells. Tumor cells were incubated with DCA (25 mM) for several time points. After either 48 h or 72 h, LT (1000 ng/mL) was added to cells for another 24 h. Results are represented as mean +/− SD of at least two different experiments with triplicate determinations for each cell line. * *p* < 0.05; ** *p* < 0.01; *** *p* < 0.001. DCA: dichloroacetate.

Results are presented in Figure 1. As can be seen, DCA was able to exert a combination effect with LUV-TRAIL in almost all cases, affecting cell proliferation and inducing apoptosis in the tumor cell lines tested. DCA alone, at this concentration, was not toxic to cells, except for MCF7 cells, where cell growth was reduced as much as 40% at 96 h (Figure 1E) and cell death reached almost 40% at this incubation time (Figure 1F). In the case of LUV-TRAIL, A549 showed the highest sensitivity, with a mean detection of 51% annexin-V^+^ cells (Figure 1B). HT29 and MCF7 cell lines did not prove to be especially sensitive to LUV-TRAIL alone (Figure 1D,F). However, when both drugs were combined, cell death was significantly increased in comparison with treatments alone, surpassing 60% of annexin-V^+^ cells in all tumor lines: ~70% in A549 cells, 60% in HT29 cells, and 80% in MCF7 cells (Figure 1B,D,F). Concomitant with these results, cell growth also significantly decreased in most cases when both drugs were combined (Figure 1A,C,E).

### 3.2. DCA Synergizes with LUV-TRAIL at Lower Doses

After finding synergy between DCA and LUV-TRAIL at the times and doses described, we sought to reproduce these results at the lowest incubation time and lowest DCA concentration, in order to achieve clinically relevant concentrations without secondary effects [51,52,53].

**Figure 2:**
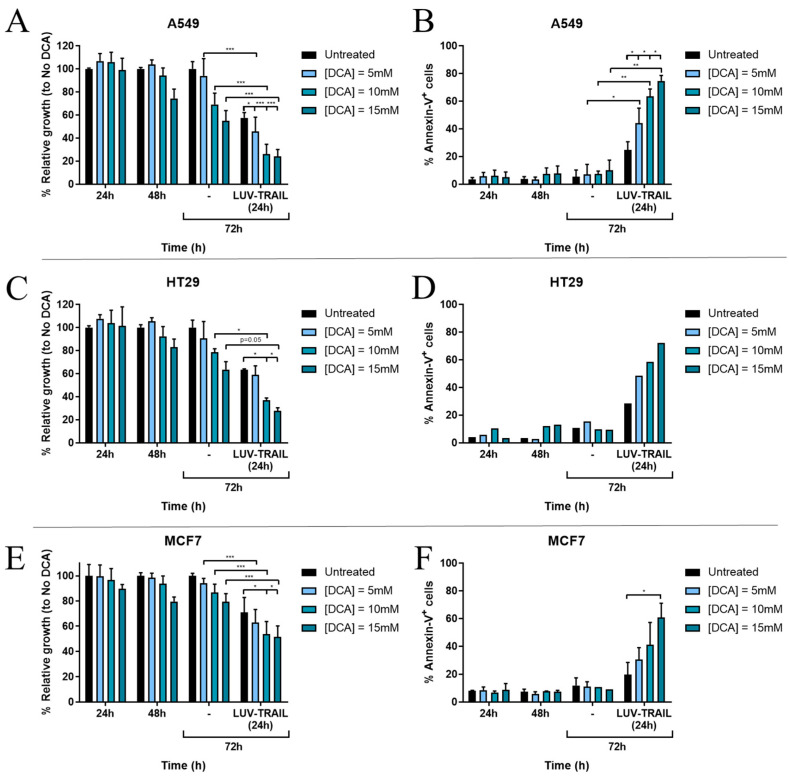
Effect of DCA + LUV-TRAIL (LT) combination in cell growth (MTT assay) and cell death (annexin-V staining and detection by flow cytometry) in (**A**,**B**) A549, (**C**,**D**) HT29, and (**E**,**F**) MCF7 cells. Tumor cells were incubated with DCA (5–15 mM) for several time points. After 48 h, cells were added LT (1000 ng/mL) for another 24 h. Results are displayed as mean +/− SD of *n* = 1–6. * *p* < 0.05; ** *p* < 0.01; *** *p* < 0.001.

Therefore, we repeated the combination treatment, performing a dose-response of 5–15 mM DCA, and using incubation times of 48 h of DCA +/− LUV-TRAIL for another 24 h. At these lower concentrations, the relative toxicity of DCA found previously on MCF7 cells was no longer observed (compare Figure 1F with Figure 2F). Cell growth and apoptosis induction results showed that DCA achieved to sensitize cells to LUV-TRAIL treatment in A549 cells, detecting significant differences in all conditions analyzed, including those at the lower DCA concentration of 5 mM (Figure 2A,B). The same tendency was observed for HT29 and MCF7 cells (Figure 2C–F).

### 3.3. DCA + LUV-TRAIL Induce Apoptosis in A549 Cells

Next, as A549 cells were the cell line with the most consistent results, we selected this tumor cell line to depict the contribution of the intrinsic or the extrinsic pathway of apoptosis in DCA + LUV-TRAIL-induced cell death. To that end, cells were incubated with DCA for 48 h, using a concentration range of 5–25 mM, followed by addition of LUV-TRAIL for 24 h, and cell death was detected by Annexin V-7-AAD staining. In addition, in some cases cells were also incubated with either the pan-caspase inhibitor z-VAD-fmk or with the specific TRAIL blocking antibody RIK2.

**Figure 3:**
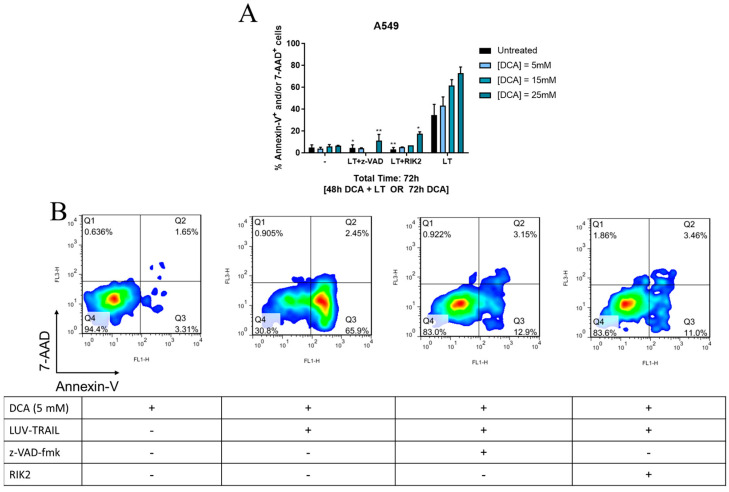
Effect of pan-caspase inhibitor z-VAD and the mAb RIK2 in DCA + LUV-TRAIL (LT)-induced cell death in A549 cells. Tumor cells were incubated with DCA (5–25 mM) for 48 h. Afterwards, LT (1000 ng/mL) was added for another 24 h. z-VAD (30 μM) or RIK2 (30 μM) were used 1 h prior to LT exposure. Cell death (by annexin-V-FITC and 7-AAD staining and detection by flow cytometry). Results are represented as mean +/− SD of at least 2 different experiments with duplicate determinations. Significances (*) referred to the experimental point vs the same column in LT alone. * *p* < 0.05; ** *p* < 0.01. (**B**) Representative dot plots of Annexin-V-FITC vs. 7-AAD staining in A549 cells, corresponding to experimental points included in (**A**). DCA: dichloroacetate.

As shown in Figure 3A, either z-VAD-fmk or RIK2 were able to almost completely abrogate cell death induced by either LUV-TRAIL or the combination of LUV-TRAIL and DCA. As it can be seen, LT induced 47% of cells positive for annexin-V, while almost no cells positive for 7-AAD were detected (Figure 3B). This clearly points out a bona fide apoptotic type of cell death induced by DCA + LUV-TRAIL, at least in this cell line.

### 3.4. Expression of c-FLIP Is Altered in a Cell-Dependent Manner after DCA Exposure

**Figure 4:**
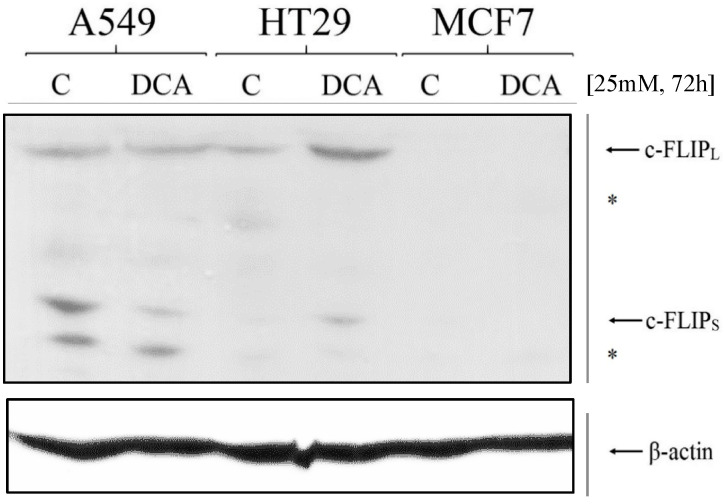
c-FLIP expression after DCA exposure. A549, HT29, and MCF7 cells were incubated for 72 h with DCA (25 mM) and then c-FLIP levels were measured by immunoblot. Non-specific bands are indicated by an asterisk (*). DCA: dichloroacetate.

In order to ascertain the reasons behind the observed LUV-TRAIL-sensitizing ability of DCA, we first analyzed the effect of DCA on c-FLIP expression. Previous studies demonstrated that glucose deprivation can induce a decrease in c-FLIP expression [54], thus priming cells for other apoptotic stimuli. DCA, by forcing OXPHOS, prevents excessive glycolysis and the Warburg effect in cancer cells [55]. Therefore, in order to further characterize DCA-sensitizing ability, we decided to explore whether DCA was able to reduce c-FLIP expression in the cell lines tested. Similar to the previous experiment, we cultured cancer cells with 25 mM DCA for 72 h. Then, extracts from treated and untreated cells were obtained. Proteins were separated by PAGE and c-FLIP expression was analyzed y immunoblot. As it can be seen in Figure 4, DCA sensitization to LUV-TRAIL also correlates with a decrease in c-FLIP expression (both long and short isoforms) in A549 cells. Despite this, this effect could not be seen in the other two cell lines tested, finding the opposite effect in HT29 cells and no expression of these proteins in MCF7 cells (Figure 4). Regarding the extrinsic pathway of apoptosis, XIAP levels were also measured, with no variation after DCA treatment (see Figure S1).

### 3.5. The Intrinsic Apoptotic Pathway Is Altered in a Cell-Dependent Manner on Tumor Cells after DCA Exposure

**Figure 5:**
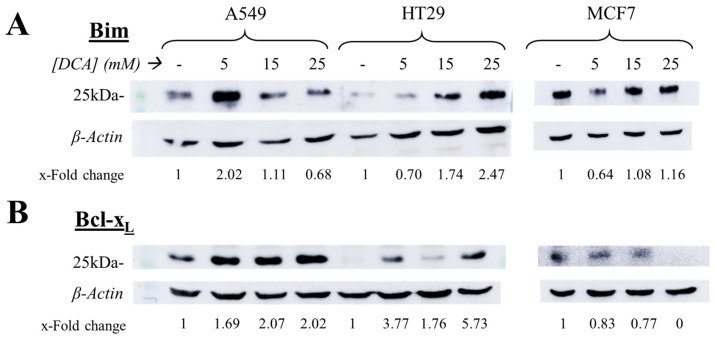
Expression of Bcl-xL (**A**) and Bim (**B**) after DCA exposure. A549, HT29, and MCF7 cells were incubated for 72 h with DCA (25 mM) and then the levels of these proteins were measured by immunoblot.

We also assessed the possible effect of DCA on priming tumor cells to the intrinsic pathway of apoptosis by analyzing the expression of Bim and also of Bcl-x_L_, one of the most relevant anti-apoptotic proteins in cancer. Tumor cells were incubated for 72 h with DCA (5–25 mM) and then, similar to the previous assay, cell extracts were obtained, proteins were separated by PAGE, and immunoblots of the specific proteins were performed. Results showed that DCA exerted different effects on tumor cells depending on the cell line tested (Figure 5). Bim expression increased in a dose-dependent way in HT29 cells, which correlates with their higher sensitivity to subsequent LUV-TRAIL treatment. However, the expression of this protein seemed to decrease in A549 cells, while in MCF7 their levels did not appear to change significantly (Figure 5). In the case of Bcl-x_L_ expression, its expression increased in A549 and HT29 cells, contrary to their higher sensitivity to DCA + LUV-TRAIL therapy. However, MCF7 cells did reduce Bcl-x_L_ levels, until no protein could be detected at the highest dose of DCA (Figure 5). Similar to the latter section, other proteins involved in the intrinsic pathway of apoptosis (Mcl-1, Bcl-2, and Bid) and caspase-3 were evaluated by immunoblot, with no correlating results (Figure S1).

### 3.6. DCA Increases DR5 Surface Expression in Tumor Cells

Next, we assessed the effect of DCA over the external expression of death receptors DR4 and DR5 in tumor cells. Thus, tumor cell lines were incubated for 72 h with DCA and expression of DR4 and DR5 was measured by flow cytometry.

**Figure 6:**
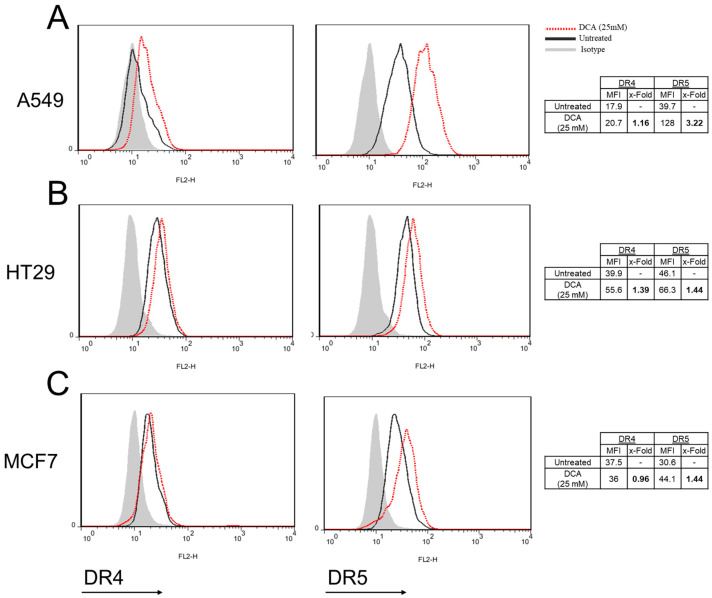
DR4 and DR5 surface expression after DCA exposure. (**A**) A549, (**B**) HT29, and (**C**) MCF7 cells were incubated for 72 h with DCA (25 mM) and then DR4 and DR5 levels were measured by flow cytometry. Bold numbers in the right tables represent the ratio between treated cells vs untreated cells (x-Fold). DCA: dichloroacetate.

As shown in Figure 6, both receptors were detected in the three tumor cell lines in basal conditions, although DR4 levels were very low in A549 and MCF7 cells (Figure 4A–C). Interestingly, expression of DR5 was clearly increased upon DCA incubation in the three cell lines, especially in the case of A549 cells (Figure 6A–C). Altogether, this increase in surface-expressed DR5 is likely the reason behind the sensitizing effect of DCA to LUV-TRAIL.

## 4. Discussion

The biologic properties of TRAIL that enable it to target malignant or infected cells while not harming normal cells make it an excellent candidate for an anti-cancer drug therapy. Unfortunately, as of today, clinical trials involving this protein have shown rather underwhelming results [56]. Several formulations have reached phase II clinical trials, such as DR4/DR5 agonistic antibodies [57], recombinant TRAIL (Dulanermin) [58,59], or other TRAIL derivatives (see NCT03298763 or [60]). However, to date, only one formulation has achieved phase III results in which the authors used a combination of Dulanermin with cisplatin and vinorelbine against non-small cell lung cancer, with limited improved potency [61]. Importantly, Dulanermin is a soluble recombinant version of TRAIL and, therefore, does not resemble the physiological transmembrane form in which cytotoxic cells mainly express TRAIL. This is in line with the poor pharmacokinetic and stability profiles of soluble TRAIL, which is most likely responsible for a weak receptor aggregation and, therefore, the decreased bioactivity of these versions of TRAIL [1]. LUV-TRAIL is a construction that, due to its high protein concentration at the liposome surface, enhances oligomerization of TRAIL receptors, inducing a higher cytotoxic action than sTRAIL in multiple types of cancer cell lines [1,21,25]. However, some cell lines remained partially resistant to this formulation.

In this work, we sought to overcome this problem by sensitizing tumor cells with the metabolic drug DCA. The results showed a clear synergistic effect, improving cell death induced by LUV-TRAIL in the three cell lines tested. This combinatory effect was further confirmed at lower doses of DCA, which are more relevant and closer to those used in clinics [62].

Death ligands, upon binding with their respective receptors, are capable of inducing either gene expression or cell death in the target cell [63]. In the specific case of TRAIL, it primarily triggers DISC/Complex I formation by recruiting FADD, caspase-8, and to a lesser extent RIPK1, cFLIPL/S, and cIAPs1/2. The main outcome of this protein complex is the activation of caspase-8, triggering the extrinsic apoptotic pathway. However, in certain circumstances, the protein complex can detach from TRAIL receptors, becoming cytosolic, in a secondary protein assembly known as Complex II, which is able to trigger necroptosis or NF-κB activation depending on the cell context [64]. Using A549 cells as a model, we sought to analyze how cells died by this treatment, by double staining with annexin-V and 7-AAD, which are apoptosis and necrosis/necroptosis markers, respectively. After LT action, most dead cells were annexin-V+ (early apoptosis), a few of them annexin-V+/7-AAD+ (late apoptosis), and 7-AAD+ only was barely detected. This confirmed that cells died by apoptosis in this combinatory treatment, drifting from early to late apoptosis as incubation time is extended. In line with these results, we found that cell death could be prevented by using either the TRAIL-blocking antibody RIK2 or by pretreatment with the caspase inhibitor z-VAD-fmk, demonstrating the cell death observed was entirely TRAIL-induced apoptosis.

This, along with the observation of DR5 up-regulation in all cell lines after DCA treatment, demonstrates that LUV-TRAIL is the main contributor to the death of these tumor cells, triggered by the extrinsic pathway activation. In addition, since z-VAD-fmk is able to block cell death, this process is caspase dependent.

In order to decipher the mechanism by which DCA sensitizes to LUV-TRAIL-induced apoptosis, we analyzed several key checkpoints controlling TRAIL-induced apoptosis, such as expression of c-FLIP, Bim, Mcl-1, or Bclx_L_, or the effect of DCA on surface expression of DR4 and DR5. Results showed divergent results within cell lines upon DCA exposure. For example, whereas c-FLIP down-regulation was only seen in A549 cells, Bim increase and Mcl-1 decrease was detected in HT29 cells, while Bcl-x_L_ expression was lower exclusively in MCF7 cells (see Figure 5, Figure 6 and Figure S1). Several groups have already assessed the effect of DCA on the expression of pro- or anti-apoptotic proteins. No change in Bcl-2, Bcl-x_L_, or Bax/Bak expression was observed in prostate cancer PC-3 cells in one study [65], while other authors have detected Mcl-1 degradation in liver cancer cells [66].

Interestingly, DCA treatment induced a strong increase in the surface expression of DR5 in the three cell lines tested. Proper activation of DR5 requires the receptor molecules to be clustered on the cell surface, which can only be efficiently achieved by membrane-bound TRAIL. In this line, we previously demonstrated that LUV-TRAIL was able to activate DR5 in a more efficient way than sTRAIL due to its capacity to contribute to DR5 clustering on the cell surface in colon cancer cells [23]. Thus, the results observed in this study correlate with our previous observations, and point towards the overexpression of surface DR5 induced by DCA as the main reason for the sensitization to LUV-TRAIL.

Mechanistically, it is not entirely clear how DCA reprograms cancer cells. It has been described that following DCA incubation, ATP production is diminished in glycolytic cancer cells, thus changing the AMP:ATP ratio and activating AMPK [43]. The AMPK complex is an energy sensor that regulates homeostasis in cells. When this enzyme is activated, it can turn on or compromise different and multiple metabolic, proliferative, and gene expression pathways in cancer [43]. Among them, AMPK is able to activate p53 [67]. On one hand, it has previously demonstrated that DCA is more effective against cancer cells with a wild type p53 [43,46]. On the other hand, other pathways independent of p53 status have been described to activate upon DCA action [68,69]. In our work, both A549 and MCF7 cells are p53 wild type, while HT29 cells possess a mutated version of this protein (https://tp53.isb-cgc.org; accessed on 13 December 2022). Still, DCA was able to synergize with LT in the three cell lines. As AMPK activation opens so many gene modulation possibilities within cells, the DCA effect can potentially affect many signaling pathways and change the expression of multiple proteins. Therefore, how or which ones will be activated or seem unlikely to depend on the specific characteristics of each tumor cell require further investigation to be identified.

DCA at 25 mM induced some level of cell death on MCF7 cells, while the other cell lines were not significantly affected by this treatment (see Figure 1). However, this induction of cell death in MCF7 cells was not observed at lower concentrations. DCA has been shown to induce higher toxicity to cells if they have defective mitochondria [34,35]. In fact, MCF7 cells have been proven to transform glutamate and also pyruvate into citrate at a lower rate than MDA-MB-231 breast cancer cells [70], and their expression of complex II, III, and V is also lower than other breast cancer cell lines [71].

Noteworthy, another relevant result to point out is the incubation time with DCA. Previous studies from our group detected cytotoxic effects of DCA in other cell lines as quickly as after 72 h of exposure [40]. Therefore, we decided to initially explore its sensitization ability by incubating the drug for 48 h or 72 h prior to LUV-TRAIL action. Interestingly, DCA was already able to synergize with LUV-TRAIL after 48 h, and as effectively as after 72 h. As shorter treatments are preferable, this can be very relevant from a clinical standpoint. In this regard, DCA is a generally well-tolerated drug, with an approximate half-life of 1 h at a dose of 50 mg/kg [72]. TRAIL, as already stated, has an excellent safety profile, as it spares normal cells.

## 5. Conclusions

In conclusion, DCA is a drug that, due to the metabolic shift that it induces in tumor cells, can sensitize cells to the cytotoxic action of LUV-TRAIL. DR5 up-regulation is the main contributor of this combinatory effect, while others seem to be tumor dependent. Therefore, energy metabolism emerges as an option for TRAIL to enhance its efficacy against resistant tumors. Obviously, additional pre-clinical in vivo experimentation is required to ascertain the benefits of this therapeutic combination and this will be one of our main goals in the near future.

## Data Availability

The datasets generated and/or analyzed during the current study are available from the corresponding authors on reasonable request.

## Supplementary Materials

The following supporting information can be downloaded at: https://www.mdpi.com/article/10.3390/jcm12020608/s1, Figure S1: Expression of multiple pro- and anti-apoptotic proteins after DCA exposure.
