# Supplementary material for: DR5 Up-Regulation Induced by Dichloroacetate Sensitizes Tumor Cells to Lipid Nanoparticles Decorated with TRAIL
Source: J Clin Med. 2023 Jan 12;12(2):608. doi: 10.3390/jcm12020608 (PMC9864242; doi:10.3390/jcm12020608)
Supplement: Supplementary file 1 [file jcm-12-00608-s001.zip › jcm-2127566-supplementary.pdf]

SUPPLEMENTAL MATERIAL

DR5 up-regulation induced by dichloroacetate sensitizes tumor cells to lipid nanoparticles decorated with TRAIL

Joaquín Marco-Brualla, Diego de Miguel, Luis Martínez-Lostao & Alberto Anel

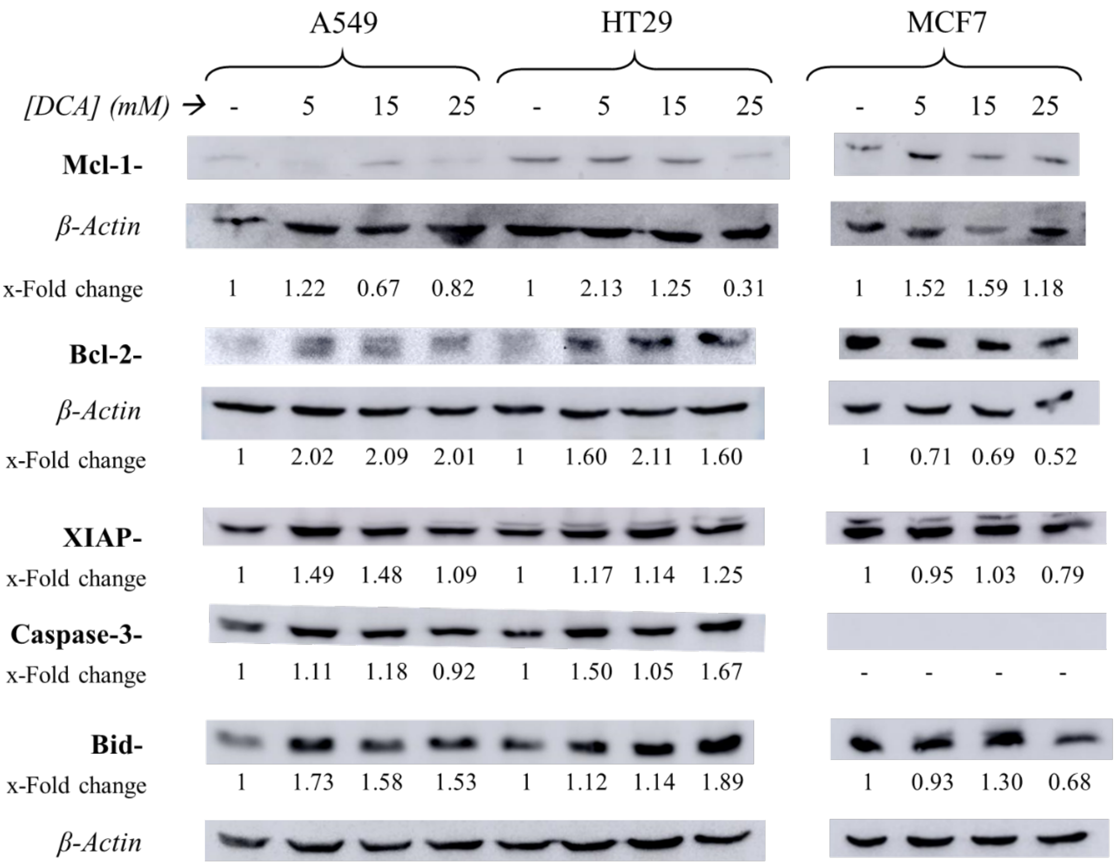

Figure S1. Expression of multiple pro- and anti-apoptotic proteins after DCA exposure. A549, HT29 and MCF7 cells were incubated for 72 h with DCA (25 mM) and then levels of Mcl-1, Bcl-2, Bid, XIAP and caspase-3 were measured by immunoblot.
